# Supplementary material for: Neonatal Red Blood Cell Transfusion Practices: A Multi-National Survey Study
Source: Healthcare (Basel). 2025 Mar 6;13(5):568. doi: 10.3390/healthcare13050568 (PMC11898768; doi:10.3390/healthcare13050568)
Supplement: Supplementary file 1 [file healthcare-13-00568-s001.zip › healthcare-3491508-supplementary.pdf]

Questionnaire tool:

Country of practice: ……………………….

Practices Characteristics:

Years of experience in NICU:

A. 1-5 years
B. 6-10 years
C. 11-20 years
D. Over 20 years

NICU practicing settings:

A. Level 1 NICU
B. Level 2 NICU
C. Level 3 NICU
D. Level 4 NICU

Duration of the PRBC transfusion:

A. 30 min
B. 1 h
C. 2 h
D. 3 h
E. 4 h

How much do you typically order for neonatal blood transfusion?

A. 10 mL/kg
B. 15 mL/kg
C. >15 mL/kg

Do you monitor the neonate's vital signs during blood transfusions even though they have not been monitored before?

A. No
B. Yes

Which vascular access do you use to transfuse red blood cells?

A. Peripheral line
B. PICC line
C. UVC
D. Peripheral line, PICC line
E. Peripheral line, UVC
F. UVC, PICC line
G. Peripheral line, UVC, PICC line

Do you use a filter for the administration of red blood cells?

A. No

B. Yes

Do you have written guidelines for neonatal blood transfusion in your unit?

A. No
B. Yes

If you answered (Yes) in the previous question, was the blood transfusion threshold stated in the guidelines either using haemoglobin or haematocrit?

A. No
B. Yes

Blood transfusion triggers

In three days old, preterm born at 24 weeks of gestation, intubated on maximum ventilatory support, Fio2 70%:

A. ≤ 10 gm/dl
B. ≤ 12 gm/dl
C. ≤ 15 gm/dl
D. No specific threshold

In three days old preterm born at 24 weeks, stable on minimal ventilation setting, Fio2 21%:

A. ≤ 10 gm/dl
B. ≤ 12 gm/dl
C. ≤ 15 gm/dl
D. No specific threshold
